# Flexible conformable hydrophobized surfaces for turbulent flow drag reduction

**DOI:** 10.1038/srep10267

**Published:** 2015-05-15

**Authors:** Joseph C Brennan, Nicasio R Geraldi, Robert H Morris, David J Fairhurst, Glen McHale, Michael I Newton

**Affiliations:** 1 School of Science and Technology, Nottingham Trent University, Clifton Lane, Nottingham, NG11 8NS, UK; 2 Faculty of Engineering & Environment, Northumbria University, Ellison Place, Newcastle upon Tyne, NE1 8ST, UK

## Abstract

In recent years extensive work has been focused onto using superhydrophobic surfaces for drag reduction applications. Superhydrophobic surfaces retain a gas layer, called a plastron, when submerged underwater in the Cassie-Baxter state with water in contact with the tops of surface roughness features. In this state the plastron allows slip to occur across the surface which results in a drag reduction. In this work we report flexible and relatively large area superhydrophobic surfaces produced using two different methods: Large roughness features were created by electrodeposition on copper meshes; Small roughness features were created by embedding carbon nanoparticles (soot) into Polydimethylsiloxane (PDMS). Both samples were made into cylinders with a diameter under 12 mm. To characterize the samples, scanning electron microscope (SEM) images and confocal microscope images were taken. The confocal microscope images were taken with each sample submerged in water to show the extent of the plastron. The hydrophobized electrodeposited copper mesh cylinders showed drag reductions of up to 32% when comparing the superhydrophobic state with a wetted out state. The soot covered cylinders achieved a 30% drag reduction when comparing the superhydrophobic state to a plain cylinder. These results were obtained for turbulent flows with Reynolds numbers 10,000 to 32,500.

When a droplet of water comes into contact with a superhydrophobic surface, it makes a contact angle of over 150° and has low hysteresis between advancing and receding contact angles. Nature has found multiple uses for superhydrophobic surfaces ranging from the self-cleaning effect of Lotus leaves^1^,^2^ to the underwater respiration of Fishing Spiders^3^,^4^. In recent years there has been significant research into using superhydrophobic surfaces for large area drag reducing applications. Much of this work has focussed on either cylinders or on flat surfaces. This work has been performed in two main ways: theoretically using computational fluid dynamics (CFD) simulations^5^,^6^,^7^,^8^,^9^,^10^,^11^,^12^,^13^,^14^ and experimentally with the aid of towing tanks and circulation experiments^15^,^16^,^17^,^18^,^19^,^20^,^21^,^22^,^23^,^24^. When an object travels through a fluid, for example a ship traveling through water, or a fluid travels through an object, for example water through a pipe, drag forces are experienced. Form drag occurs due to the physical dimensions of the object obstructing and altering the flow of the fluid. Skin friction also occurs due to the fluid traveling over the surface of the sample. The hydrodynamic property of this interaction leads to a characteristic parameter, the drag coefficient. The drag coefficient of an object is a dimensionless quantity that describes the drag force that an object of a certain shape experiences whilst travelling through a fluid. A high drag coefficient shows that there is a large drag force on the object, whereas a low drag coefficient shows that there is a low drag force on the object. By using superhydrophobic surfaces the amount of skin friction that the object being tested experiences can be reduced. Superhydrophobic surfaces can exist in one of two extremes. The Cassie-Baxter state describes a superhydrophobic surface where the surface roughness features do not penetrate into the fluid that the surface is submerged in. The other extreme is the Wenzel state where the fluid fully surrounds all of the roughness features. This Wenzel state is not superhydrophobic due to lower contact angles achieved when the sample is in this state. As well as potentially superhydrophobic surfaces being in one of these two states the surface can also be in an intermediate state between the two. In this state the surface is partially wetted so that it is neither in the full Cassie-Baxter state nor the full Wenzel state. For a superhydrophobic surface to retain a plastron layer, the sample needs to be in the Cassie-Baxter state or at least in the partially wetted state. Superhydrophobic surfaces are able to achieve a drag reduction due to the presence of the plastron layer and the large slip that can occur across the surface of this plastron layer^9^,^23^,^25^. The air layer has a much lower viscosity than the fluid so acts to lubricate the motion of the fluid. If the superhydrophobic surface is in the Wenzel state then the sample is just a roughened surface which leads to a drag increase when there is not a large increase in Reynolds number value. The drag reduction lifespan of a surface is limited by the length of time for which the sample remains in the Cassie-Baxter state^26^. The plastron layer can be removed in two main ways, through diffusion of the gas layer into the surrounding fluid or by being stripped by the relative movement between the surface and the fluid. When a surface becomes fully wetted in one area, it can rapidly become fully wetted over the whole surface if there is no specific surface roughness designed to stop this behaviour. Recently there has been work into methods of retaining and replenishing the plastron air layer^27^,^28^,^29^. This work negates some of the plastron lifetime problems that samples currently experience by using the Leidenfrost effect to create a water vapour layer around the sample or by using electrolysis to create a hydrogen layer between surface features.

Relatively large area superhydrophobic surfaces have been created before. These are usually achieved through tiling^30^,^31^, painting^32^ or spraying^33^ a superhydrophobic product onto a surface. The main issue with tiling is making sure that the tiles are placed perfectly level on the surface. A small misalignment can lead to extra drag that hides the underlying effect of the superhydrophobic surfaces being tested. Painting and spraying superhydrophobic surfaces create large areas that are uniform and level but both have issues with the durability of the superhydrophobic layer that they create. This leads to a shorter product lifetime than other methods for creating superhydrophobic surfaces.

Drag coefficient values for smooth cylinders have been widely researched. In the Reynolds number range presented in this work, 10,000 to 32,500, the drag coefficient value for a plain cylinder is ~1.1^34^. The effect that roughness has on the drag coefficient of a cylinder has also been widely researched^35^,^36^,^37^,^38^,^39^. The roughness of a cylinders surface can be quantified using the roughness parameter
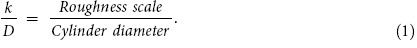


Previously, confocal microscopy has been used to quantify the thickness of plastron air layers on superhydrophobic surfaces^26^,^40^. Confocal microscopy has also been used to take images of the coverage of the plastron layer on the superhydrophobic surfaces^40^. Confocal microscopes are able to measure the thickness of a plastron layer by focusing on the substrate, the reflection that occurs at the gas-liquid interface and on the top of the roughness features. This technique only works for surfaces that reflect light. By using the known position of the sample surface and the gas-liquid interface, the thickness of the plastron and the protrusion height of the sample into the fluid can be measured simultaneously.

In this report we outline two methods for creating large area flexible superhydrophobic surfaces. These are an electrodeposition method for creating large area superhydrophobic copper mesh samples and a method for making large area superhydrophobic soot samples. The combination of these two methods allows surfaces with various feature heights and spacings to be made. For the drag coefficient experiments, we use a recirculating flow chamber with a Laser Doppler Anemometry (LDA) system. This LDA system is used to measure water velocities downstream of the superhydrophobic sample being tested. As well as these drag coefficient results, the superhydrophobic surfaces are characterized using confocal microscopy and SEM. All experiments were undertaken with the sample underwater for under an hour. This has been shown to negate the effect of diffusion of the plastron air layer into the surrounding fluid^26^. By negating the effects of diffusion, the stripping of the plastron layer of our samples is completely determined by the shear stress that the flow of fluid has on the surface. Prior to confocal microscopy, the samples were subject to a flow of water that caused a shear stress on the surface to remove some of the plastron layer as was experienced by the samples being tested for drag coefficient values. For drag reduction values the electrodeposited copper mesh samples show drag reduction results when comparing a plastron-bearing superhydrophobic surface to the same surface without the plastron air layer. The soot sample results show a drag reduction when comparing the plastron-bearing surface to a plain cylinder. This is due to the fact that wetting out the superhydrophobic soot surface with a low surface tension solvent is not possible without damaging the underlying surface. The results presented in this work show that both methods of creating superhydrophobic samples lead to surfaces with drag reductions of up to 32% when comparing the plastron bearing samples to the same sample after the plastron air layer has been removed and that this drag reduction is caused through a combination of plastron thickness, plastron surface coverage and protrusion length of surface roughness features in to the fluid.

## Results

**Figure 1:**
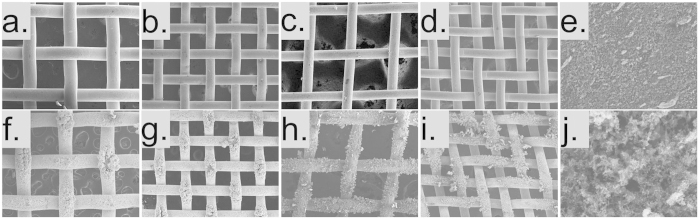
SEM images of copper mesh, electrodeposited copper mesh and soot surface. (**a**-**d**) Plain copper meshes, (**e**) low magnification soot surface, (**f**-**i**) electrodeposited copper meshes and (**j**) high magnification soot surface.

SEM images of plain copper meshes, electrodeposited copper meshes and soot surfaces are shown in Fig. 1(a–j). Figure 1(a–d) shows plain meshes with mesh numbers 40, 60, 100 and 200 respectively. The wire diameter and spacing for each mesh number can be found in Table 1. The repeating pattern distance measurements shown in Table 2 were also measured using the SEM images.

Figure 1(f–i) shows the electrodeposited copper meshes in the same order. The effect of the copper electrodeposition can be seen when comparing the plain copper meshes Fig. 1(a–d) to the electrodeposited copper meshes Fig. 1(f–i). Two magnifications of soot surface are included showing the large scale roughness of the piled up soot particles (Fig. 1(e)) and a higher magnification showing the soot surface (Fig. 1(j)). Figures 1a,b,f,g were taken at x50 magnification. Figures 1c,d,h,i were taken at a magnification of x140. Figures 1e,j were taken at magnifications of x400 and x4000 respectively.

**Figure 2:**
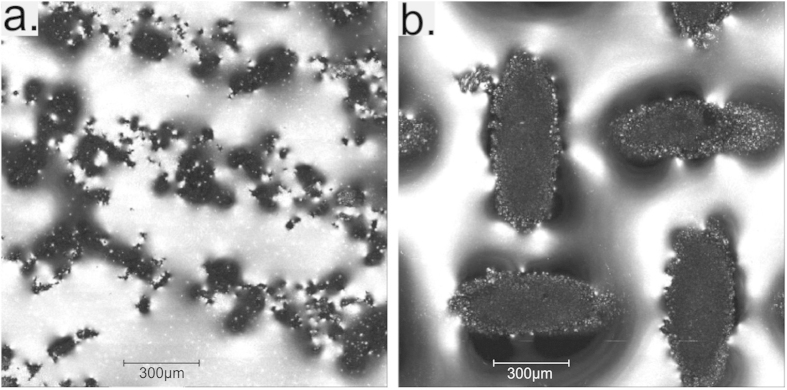
In both images the light areas show the reflective plastron-water interface. (**a**) Soot covered sample. (**b**) 40 mesh number electrodeposited copper mesh with wire diameter of 221 μm and wire spacing of 490 μm.

All surfaces were characterised using advancing and receding contact angles. For this, images were taken and analysed using a drop shape analysis system (Krüss DSA10). Surface feature height, plastron thickness, protrusion of features into the liquid and plastron coverage was measured using a confocal microscope (Leica SP5C Spectral Confocal Laser Scanning Microscope). Due to the low reflectivity of the matt black soot surface we were unable to get images of the surface using the confocal microscope, but were able to get images of the plastron air layer coverage. Samples were imaged after water was flowed over the surface. This is to replicate the conditions they face in the water circulating flow chamber where drag coefficient measurements were taken. The same flow process was undertaken as has been previously published by Brennan *et al.*^24^. Results from the contact angle measurements and confocal microscope are shown in Table 2. Figure 2 shows images taken using the confocal microscope of a soot sample and the 40 mesh number electrodeposited sample with wire diameter of 221 μm and spacing between wires of 490 μm. These images are a combination of images taken from a confocal stack that have been combined to show the reflective plastron air layer and the protruding parts of the samples. The confocal microscope images do not show the entirety of the plastron air layer. This is because where the plastron meets the surface, the water forms a meniscus which reflects light away from the detector. This results in the images under representing the extent of the plastron coverage. The solid fraction (δ) was measured for each of the electrodeposited copper mesh surfaces using the confocal microscope images.

The results for advancing contact angles are similar for all surfaces being tested. Each has an advancing contact angle of over 150°. The soot coated surface has a high advancing contact angle of 159 ± 2° and a high receding angle of 153 ± 2° resulting in a superhydrophobic surface with low hysteresis. The electrodeposited copper mesh samples have high contact angles but with receding contact angles much lower resulting in large hysteresis of up to 25°.

All drag coefficient measurements were taken in a large water circulating flow chamber with the cylinders placed at a depth of 1.2 m. At this depth it has been previously shown that the plastron air layer has a lifetime of over 3 hours^26^. A motor with variable speeds produced laminar flow with water velocities in the range of 1.4 ms^-1^ to 2.8 ms^-1^ allowing control of the Reynolds number experienced by the samples. Water velocity measurements were taken tangentially across the cylinder samples and drag coefficient values were calculated from these velocities using the momentum deficit technique^41^. For the momentum deficit technique to be used with a 1D LDA system the LDA measurement volume needs to be 30 cylinder diameters downstream of the cylinder being tested. At this point the vortexes formed by the cylinder are reduced such that the water velocity perpendicular to the cylinder is negligible. The LDA took measurements every 3 mm from one side of the cylinder to the other. The total distance was set so that free-stream water velocities could be found either side of the cylinder. A Gaussian was fitted to the raw LDA data allowing the drag coefficient to be calculated using^41^
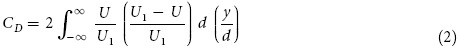
where 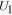 is the free stream velocity, 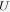 is the velocity at a given point as measured by the LDA, 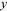 is the horizontal displacement and 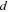 is the diameter of the cylinder being tested.

To investigate the effect that the plastron air layer has on the drag coefficient of the cylinder being tested the samples were run in the Cassie-Baxter state with the plastron air layer present between surface roughness features and in the Wenzel state where there is no plastron air layer present on the surface of the sample. To achieve this Wenzel state without the plastron air layer the copper mesh coated cylinders were dipped in ethanol^15^ which prevents the build-up of the plastron air layer. When an ethanol pre-treatment was applied to the soot covered samples the plastron layer was not fully prevented and resulted in partial destruction of the soot layer.

**Figure 3:**
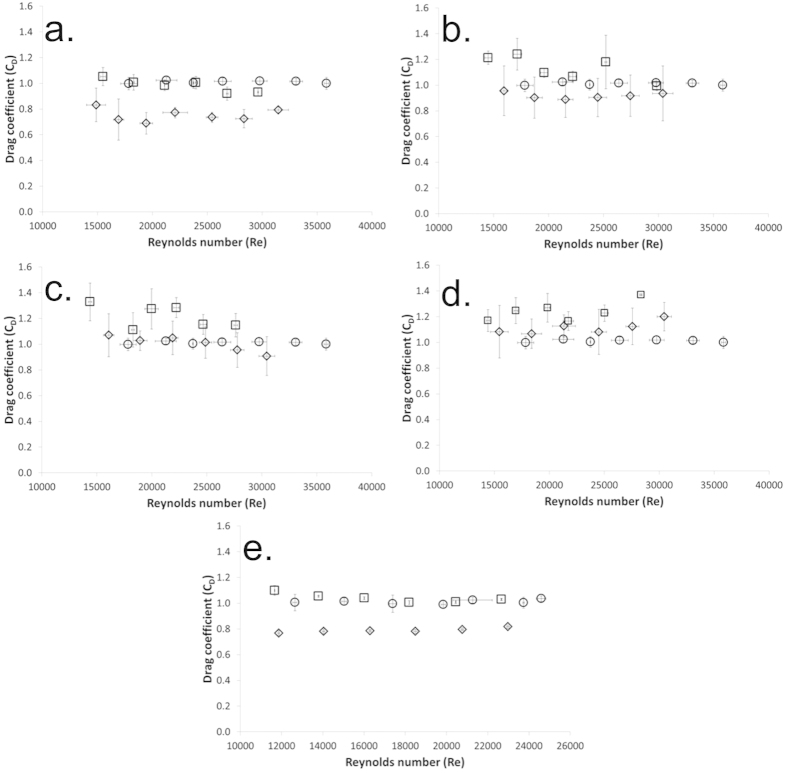
Drag coefficient (C_D_) values at various Reynolds numbers (Re) for all superhydrophobic cylinder samples. The graphs show plastron bearing results (diamonds), wetted out after pre-treatment results (squares) and smooth plain cylinder (circles) for (**a**) electrodeposited copper mesh number 40 with wire diameter 221 μm and spacing of 490 μm, (**b**) electrodeposited copper mesh number 60 with wire diameter 195 μm and spacing of 265 μm, (**c**) electrodeposited copper mesh number 100 with wire diameter 59 μm and spacing of 210 μm, (**d**) electrodeposited copper mesh number 200 with wire diameter 55 μm and spacing of 75 μm and (**e**) soot.

Figure 3(a–e) shows the average results for drag coefficient against Reynolds number for all 5 superhydrophobic samples in their plastron-bearing state and after the wetting out pre-treatment that prevents the build-up of the plastron air layer. The graphs also show results from a smooth plain cylinder. Each graph shows results for multiple cylinders tested a minimum of 5 times each in the plastron-bearing state and in the wetted out state after pre-treatment.

All electrodeposited copper mesh samples show a drag reduction when comparing the plastron bearing runs to the same sample wetted out with an ethanol pre-treatment. For the soot covered sample it is better to compare the plastron bearing state to that of a plain cylinder. This is due to the ethanol wetting out pre-treatment damaging the surface of the sample. The highest drag reduction values are 32% for the 40 mesh number electrodeposited copper mesh with wire diameter of 221 μm and spacing of 490 μm and 30% for the soot covered sample. The smallest error on the drag coefficient value is for the soot covered cylinder. This shows that the smoother surface obtained through that manufacturing process leads to more repeatable surface when compared to the electrodeposited copper mesh surfaces. These drag reduction percentages for this geometry are similar to previously published results^24^ where drag reductions of 28% were achieved on cylinders in the same Reynolds number range as tested here. CFD simulations have also been conducted on cylinders^42^,^43^ and have shown the effect of slip on cylinders. Other superhydrophobic geometries have led to drag reductions of 40% in microchannels^12^,^44^ and 66% in a rheometer set-up^25^.

Previous work has shown no direct correlation between contact angles and slip lengths^45^. For this reason it is most appropriate to look at the coverage of the plastron on the surface of the samples and the size of the repeating pattern (L) on the electrodeposited copper mesh samples. Confocal microscope images of the soot covered samples show that they have a maximum distance of approximately 1 mm between points where the soot is in contact with the liquid. The repeating pattern size for the electrodeposited copper mesh can be found from the SEM images in Fig. 1.

It has been previously shown that the slip length for ridge geometries can be estimated using^46^;
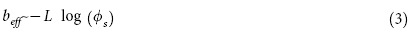
Where 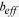 is the effective slip length, L is the roughness periodicity and Φ_s_ is the solid fraction.

The effective slip length for a regular repeating array of posts can be estimated using^46^;
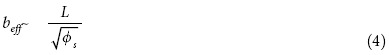
For an array of regularly spaced circular pillars the effective slip length can be found using^47^;
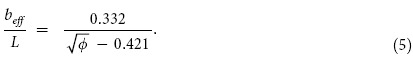


The electrodeposited copper mesh coated cylinders investigated in this report can be considered to be made up of a combination of pillars and ridges. The effective slip length for an array of pillars and for ridges is proportional to L. This means as the repeating geometry size increases so does the effective slip length. The drag reduction results shown in Fig. 3 show that as the repeating pattern size increases so does the drag reduction value for the electrodeposited copper mesh samples.

All of the cylinders tested were made with similar diameters and were tested across the same range of Reynolds numbers. This means that all of the samples had similar values for boundary layer thickness. As Reynolds number increases the boundary layer thickness decreases^48^ which in turn should lead to a larger drag reduction caused by the plastron bearing samples. This is not seen in this work as our results show a fairly constant value of drag reduction as Reynolds number increases for the plastron bearing samples. Since Reynolds number values are related to the speed of the water this discrepancy is most likely due to a drag increase caused by the greater shear stresses on the plastron layer causing some of it to be removed from the samples.

## Discussion

We have shown two methods for creating large area flexible superhydrophobic surfaces. These have been created through the electrodeposition of copper on copper meshes with various wire diameters and spacing and by creating flexible PDMS surfaces with soot embedded into the surface. Both hydrophobized copper mesh and soot can be used to create drag reductions of up to 32%. The copper mesh samples reach this 32% drag reduction value when comparing the plastron-bearing sample to the same sample wetted out with an ethanol pre-treatment that prevents the build-up of the plastron air layer. There is not always a drag reduction when comparing the plastron bearing electrodeposited copper meshes to a plain rod. The samples made from the electrodeposited copper mesh with a wire diameter of 221 μm and spacing of 490 μm and electrodeposited copper mesh with a wire diameter of 195 μm and wire spacing of 265 μm both show drag reductions when comparing the plastron bearing runs to the runs with the plain cylinders. However the samples made with electrodeposited copper mesh with a wire diameter of 59 μm and spacing of 210 μm in the plastron bearing state have drag coefficient values that are the same as a plain rod. The samples made with the electrodeposited copper mesh with a wire diameter of 55 μm and spacing of 75 μm in the plastron bearing state have a drag increase compared to a plain rod even with the plastron air layer present. The soot samples achieve a drag reduction of up to 30% when comparing a superhydrophobic sample to a plain smooth cylinder. The soot covered sample is compared to a smooth cylinder due to the inability to fully wet out the surface without damaging it during the process. These drag reduction results show that the large plastron coverage obtained by the soot surface leads to as high a value of drag reduction as the thick plastron obtained using the largest copper mesh tested which had wire diameters of 221 μm and spacing between wires of 490 μm. These results show that a large drag reduction can be obtained through large plastron coverage as shown by the hierarchical structures formed by the clumping of soot particles on the surface of the sample and by the hierarchical structure of the small copper depositions on the large copper mesh. These experiments have reduced the effect of plastron lifetime constraints by keeping the time of the sample being submerged underwater to under 1 hour. This leads to negligible diffusion of the plastron into the water. Overcoming this is one of the main challenges currently faced when using superhydrophobic surfaces for drag reduction applications.

## Methods

Superhydrophobic cylinders were created via two different methods. Superhydrophobic copper mesh samples were created by electrodeposition of copper onto copper meshes with various wire diameters and spacing. This electrodeposited copper mesh was then hydrophobized and wrapped around a cylinder to make a superhydrophobic covered cylinder. Soot covered samples were created by partially embedding soot into Polydimethylsiloxane (PDMS) and then wrapping this superhydrophobic surface around a cylinder to again make a superhydrophobic cylinder.

To create superhydrophobic copper mesh samples four copper meshes of various wire diameters and spacing were chosen to have copper electrodeposited onto their surface. These meshes had mesh numbers of 40, 60, 100 and 200. A mesh number is how many holes there are in an inch across the surface of the mesh. This means that a high mesh number mesh has a thin wire with small spaces whereas a low mesh number mesh has thicker wires with wider spacing. The wire diameter and spacing for each mesh are shown in Table 1.

Superhydrophobic surfaces can be made through electrodeposition of copper onto a copper substrate using a copper-acid bath method that gives diffusion limited aggregation^49^,^50^,^51^. The solution used was made from 1.25 M copper sulphate (Copper II sulphate hydrate 98%, Sigma-Aldrich) in 0.26 M sulphuric acid (SG 1.84, 98+%, Fisher). A total of 1 L was used for each electrodeposition and the solution was changed after two samples to avoid contamination. Before electrodeposition could commence, the copper meshes were bonded to a clean piece of copper using a cyanoacrylate adhesive. The edge of the copper mesh and any areas of the exposed copper sheet were then coated in nail varnish to act as an electrical insulator. This combined sheet was then bent around a steel cylinder with a diameter of 20 cm. This has the effect of improving the flatness of the copper mesh and improving the electrical contact between the mesh and the copper sheet. This bent sheet is used as the cathode during electrodeposition. The anode is made from a copper sheet twice the size of the cathode sheet and is bent around the same 20 cm diameter steel cylinder so that all points of cathode surface are equidistant from the anode surface. The cathode and anode were held 1 cm apart for the duration of the electrodeposition using acrylic spacers. Electrodeposition occurred at a current density of 240 mAcm^-2^ for all meshes and lasted for 30 minutes. The copper mesh was then rinsed and removed from the copper backing sheet to leave a copper mesh with growth features on the surface. The mesh was then hydrophobized using a solution of 5% commercial waterproofing product (Granger’s Extreme Wash In) and 95% warm water. The mesh was then placed in an oven at 60 °C for 4 hours to evaporate any water and solvent from the waterproofing product from the surface leaving a superhydrophobic copper mesh.

To create the superhydrophobic mesh cylinders the hydrophobized electrodeposited mesh was wrapped around an acrylic pipe with an outer diameter of 10 mm and an inner diameter of 8 mm. This pipe had been pre-cut to the right length using a laser cutter (Universal M-300) and a 2 mm slit was also cut into the acrylic pipes. This slit was there so that when the mesh was being wrapped around the pipe both ends could be inserted into the slit and the mesh could be folded around the acrylic to keep the mesh tight and flat on the surface. Two part epoxy glue (Araldite 2029) was then used to glue the two ends of the copper mesh together above the slit in the acrylic. To add stability, the acrylic tube and mesh was attached to a 6 mm diameter brass core using acrylic pieces. This brass core could then be attached to two end pieces of brass that were machined so that they could be held in the large recirculating flow chamber used to measure drag coefficients. Small superhydrophobic samples were made as well as superhydrophobic copper mesh cylinders. These flat samples were electrodeposited and hydrophobized in the same way as the cylinder meshes but were cut into smaller pieced and attached to flat microscope slides. These samples were made so that properties such as contact angle, plastron thickness and plastron coverage could be measured.

To create a superhydrophobic flexible surface with small feature height and large area coverage we used a method similar to Geraldi *et al.*^52^. The method was modified so that larger areas of PDMS, approximately 150 mm by 50 mm, were coated in soot in order to produce flexible superhydrophobic films with bi-scale roughness. The soot produces nanoscale roughness and as the particles build up on the surface during the fabrication process, a network structure forms that produces micro-scale roughness^53^. Rectangular areas were prepared, with widths equal to the circumference of the 10 mm diameter brass cylinders, to which the flexible surfaces were mounted. Using a cyanoacrylate adhesive and primer, the soot coated PDMS sections were glued to the cylinder, making sure the edges of each section were aligned accurately to minimise the effects of the seams.

Both sample making processes created a join along the length of the cylinder where the superhydrophobic surface joins onto itself. In each case this is minimised by careful levelling and cutting of the surfaces. To minimise the effect this has on the drag coefficient results the join was placed on the downstream side of the sample. This places it behind the separation point reducing its impact.

**Figure 4:**
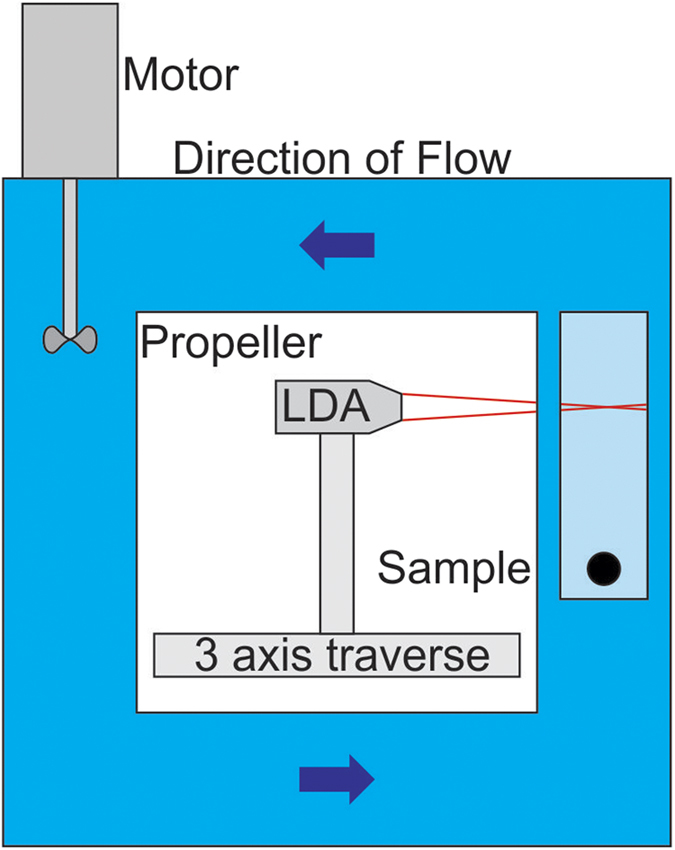
Simple schematic of the water circulating flow chamber and Laser Doppler Anemometer (LDA) setup used to measure drag coefficients of the various superhydrophobic cylinders.

A simple schematic of the experimental setup used for measuring the drag coefficient of the superhydrophobic cylinders is shown in Fig. 4. The velocity of the water downstream of the sample was measured using a Laser Doppler Anemometer (Dantec dynamics flow explorer) (LDA). The LDA was attached to a 3 axis traverse which could move the laser so that it measured far enough away on either side of the sample that free stream water velocities could be found. Between these free stream values, the LDA took a velocity reading every 3 mm. All cylinders were tested in a plastron bearing state and after a pre-treatment that prevented the build-up of the plastron air layer. To achieve this state without the plastron air layer, both the copper mesh coated cylinders and the soot coated cylinders were dipped in ethanol^15^. By preventing the build-up of the plastron air layer we are able to compare the same sample with and without the plastron to test the effect that the plastron has on drag reduction.

## Author Contributions

J.C.B and N.R.G prepared the samples, performed the experiments and analysed the results. R.H.M wrote the programme that was used to analyse the results. D.J.F, G.M and M.I.N conceived the experiment. J.C.B wrote the manuscript. All authors reviewed the manuscript.

## Additional Information

**How to cite this article**: Brennan, J. C. *et al.* Flexible conformable hydrophobized surfaces for turbulent flow drag reduction. *Sci. Rep.* **5**, 10267; doi: 10.1038/srep10267 (2015).

## Figures and Tables

**Table 1:** Mesh numbers and physical sizes for the 4 meshes used in this report.

**Mesh number**	**Wire diameter (μm)**	**Wire spacing (μm)**
40	221 ± 9	488 ± 23
60	165 ± 5	263 ± 11
100	59 ± 1	208 ± 4
200	54.5 ± 0.7	77 ± 6

**Table 2:** Contact angles, feature thickness, plastron thickness, feature protrusion heights, repeating pattern length and solid fraction.

**Copper mesh number or soot**	**Advancing contact angle (°)**	**Receding contact angle (°)**	**Roughness parameter (roughness scale/cylinder diameter)**	**Average feature thickness (μm)**	**Average plastron thickness post water flow (μm)**	**Average feature protrusion height post water flow (μm)**	**Repeating pattern length (L) (μm)**	**Solid fraction (%)**
40	161 ± 7	140 ± 11	0.049 ± 0.001	550 ± 9	506 ± 12	44 ± 3	709 ± 9	35 ± 5
60	158 ± 3	133 ± 2	0.034 ± 0.001	378 ± 7	283 ± 16	95 ± 9	428 ± 6	55 ± 6
100	163 ± 2	141 ± 3	0.013 ± 0.001	131 ± 7	103 ± 12	28 ± 5	267 ± 5	27 ± 4
200	152 ± 2	136 ± 1	0.0085 ± 0.0008	92 ± 6	64 ± 10	28 ± 4	132 ± 3	39 ± 9
Soot	159 ± 2	153 ± 2	n/a	n/a	n/a	n/a	n/a	n/a
